# A syndemic of inequitable gender norms and intersecting stigmas on condom self-efficacy and practices among displaced youth living in urban slums in Uganda: a community-based cross-sectional study

**DOI:** 10.1186/s13031-023-00531-y

**Published:** 2023-08-20

**Authors:** Moses Okumu, Carmen H. Logie, Anissa S. Chitwanga, Robert Hakiza, Peter Kyambadde

**Affiliations:** 1 School of Social Work, University of Illinois, Urbana-Champaign, 1010 W. Nevada St., Urbana, IL 61801 USA; 2 School of Social Sciences, Uganda Christian University, Mukono, Uganda; 3 Factor Inwentash Faculty of Social Work, University of Toronto, 246 Bloor Street West, Toronto, ON M5S 1V4 Canada; 4 United Nations University Institute for Water, Environment, and Health (UNU-INWEH), 204-175 Longwood Rd S, Hamilton, ON L8P 0A1 Canada; 5 Young African Refugees for Integral Development (YARID), Nsambya Gogonya, Kampala, Uganda; 6 AIDS Control Program, Ministry of Health, Plot 6, Lourdel Road, Nakasero, Kampala, Uganda; 7 Most at Risk Population Initiative, Mulago Hospital, Kampala, Uganda

**Keywords:** Inequitable gender norms, Intersectional stigma, Syndemics, Condom use efficacy and use, Displaced urban refugees, Uganda

## Abstract

**Background:**

Adverse socio-cultural factors compromise the implementation of HIV prevention strategies among displaced youth. While condoms are an affordable and effective HIV prevention strategy for youth, stigma and inequitable gender norms may constrain condom self-efficacy (i.e., knowledge, intentions, and relationship dynamics that facilitate condom negotiation) and use. Further, knowledge of contextually appropriate HIV prevention approaches are constrained by limited understanding of the socio-cultural conditions that affect condom self-efficacy and use among displaced youth. Guided by syndemics theory, we examine independent and joint effects of adverse socio-cultural factors associated with condom self-efficacy and use among displaced youth living in urban slums in Kampala, Uganda.

**Methods:**

We conducted a community-based cross-sectional survey of displaced youth aged 16–24 years living in five slums in Kampala. We used multivariable logistic regression and multivariate linear regression to assess independent and two-way interactions among adverse socio-cultural factors (adolescent sexual and reproductive health-related stigma [A-SRH stigma], perceived HIV-related stigma, and beliefs in harmful inequitable gender norms) on condom self-efficacy and recent consistent condom use. We calculated the prevalence and co-occurrence of adverse socio-cultural factors; conducted regression analyses to create unique profiles of adverse socio-cultural factors; and then assessed joint effects of adverse socio-cultural factors on condom self-efficacy and practices.

**Results:**

Among participants (mean age: 19.59 years; SD: 2.59; women: *n* = 333, men: *n* = 112), 62.5% were sexually active. Of these, only 53.3% reported recent consistent condom use. Overall, 42.73% of participants reported two co-occurring adverse socio-cultural factors, and 16.63% reported three co-occurring exposures. We found a joint effect of beliefs in harmful inequitable gender norms with high A-SRH stigma (β = − 0.20; *p* < 0.05) and high A-SRH stigma with high perceived HIV stigma (β = − 0.31; *p* < 0.001) on reduced condom self-efficacy. We found a multiplicative interaction between high A-SRH stigma with high perceived HIV stigma (aOR = 0.52; 95% CI 0.28, 0.96) on recent consistent condom use. Additionally, we found that condom self-efficacy (aOR = 1.01; 95% CI 1.05, 1.16) and safer sexual communication (aOR = 2.12; 95% CI 1.54, 2.91) acted as protective factors on inconsistent condom use.

**Conclusions:**

Displaced youth living in urban slums exhibited low consistent condom use. Intersecting stigmas were associated with lower condom self-efficacy—a protective factor linked with increased consistent condom use. Findings highlight the importance of gender transformative and intersectional stigma reduction approaches to increase sexual agency and safer sex practices among Kampala’s slum-dwelling displaced youth.

## Background

By the end of 2022, over 110 million people worldwide were forcibly displaced, including 27.1 million refugees [1]. About half of those refugees are less than 18 years old, meaning that a large proportion of the global refugee population are youth who are or will soon become sexually active [2]. Yet refugees in humanitarian settings face multiple barriers to maintain optimal sexual and reproductive health (SRH) care given that the majority reside in low- and middle-income countries whose healthcare systems are unable to meet the demands of their residents [3, 4]. This concerning lack of SRH services is further compounded by significant gaps in research conducted in humanitarian settings, leaving researchers with insufficient evidence to develop appropriate prevention, promotion and intervention services for these high-need populations. Notably, because SRH care for youth living in humanitarian contexts is especially scarce, their SRH needs remain largely unaddressed [5]. Indeed, a recent systematic review highlighted the deficit of SRH interventions tailored to youth in humanitarian settings [6]. This finding is particularly troubling given that youth in humanitarian contexts face increased vulnerabilities to HIV and other sexually transmitted infections (STIs) due both to the lack of adequate SRH care and the elevated experiences of sexual violence, intersecting stigmas, and poverty that characterize many humanitarian contexts [6–10]. Together, a lack of resources and a lack of knowledge continue to impede the development of effective SRH supports for displaced youth living in humanitarian settings.

With over 1.5 million forcibly displaced people, Uganda hosts the most displaced persons of any sub-Saharan Africa country and the fourth most of all countries globally [11]. Further, 62% of forcibly displaced individuals residing in Uganda are under the age of 18 years [2, 11], and about a third of the country’s forcibly displaced live in informal settlements, often slums [11, 12]. Young people living in slums face economic deprivation, poor and overcrowded living conditions, food insecurity, and violence and victimization [13–17]. Slums also display high rates of transactional sex, substance use, and sexual practices that elevate HIV and STI exposure [16, 18, 19]. For this reason, STI and HIV rates among young people living in slums tend to be higher than among those living in formal settlements. In the slums of Kampala, Uganda, 13.9% of youth are HIV positive [16]—compared to the national HIV prevalence rate of 6.5% [20]—and 26.1% report having an STI [21]. Notably, the HIV prevalence rate among adolescent girls and young women in Uganda (not only in slums) is over four times higher than the rate among adolescent boys and young men, highlighting the exceptional risk of HIV infection and STIs faced by refugee young women living in Uganda’s informal settlements [20].

Consistent condom use remains one of the most effective protective strategies against STI acquisition—including HIV—both within and beyond humanitarian contexts. UNAIDS’ 2020 report showed that in sub-Saharan Africa, only 21% of young women and 37% of young men aged 15–24 reported using external condoms during their last sexual intercourse, below the global average of 39% for young women and 51% for young men [22]. In Uganda, the vast majority of youth aged 15–24 years—85.5% of adolescent girls and young women and 87.1% of adolescent boys and young men—report knowing that wearing external condoms during sex can reduce their likelihood of contracting HIV [23]. However, previous studies have estimated that only 53–66% [21, 24, 25] of sexually active youth living in Uganda’s slums consistently use external condoms, signalling significant gaps between reported knowledge of and actual use of condoms, far below the global target of 90% by 2025 [26]. Among sexually active youth living in Kampala slums specifically, a cross-sectional survey found that only half reported using condoms [25, 27, 28]. This figure is likely not only attributable to a lack of access to condoms, but also other barriers to condom use such as cost, negative attitudes, reduced sexual pleasure, fit-and-feel problems and erection difficulties. One 2014 study among displaced youth in the slums of Gulu (another Ugandan city) found that while 63% of these slum-dwelling youth had access to condoms, only one-quarter had used a condom in during their last sexual intercourse [29]. Findings among Uganda’s adolescent refugee girls also reveal gaps in the HIV prevention cascade. In a cross-sectional study of girls living in Uganda’s Nakivale refugee settlement, participants reported a condom use rate of 18% during sex in past 3 months [30]. Clearly, identifying barriers and facilitators to condom accessibility and condom self-efficacy required to promote SRH among refugee youth, in and beyond Uganda.

### Syndemics, adverse socio-cultural factors, and condom self-efficacy and practices

Forcibly displaced youth’s increased exposure to HIV can in part be explained by a syndemics framework that spotlights how factors spanning socio-cultural, interpersonal, and behavioral levels interact to influence health outcomes [31–34]. For instance, a recent study among refugees in Uganda found that syndemic interactions between frequent alcohol use, depression, and violence experiences were associated with increased HIV exposure [35]. More broadly, a 2020 review [36] of research on syndemics associated with HIV identified 60 articles that reported co-conditions such as substance use (*n* = 40; 67%), high-risk sexual practices (*n* = 36; 60%), depression (*n* = 36; 60%), interpersonal violence (*n* = 35; 58%), stigma (*n* = 19; 32%), STIs (*n* = 16; 27%), trauma (*n* = 14; 23%), and noncommunicable diseases (*n* = 6; 10%)—many of which are prevalent among the youth in Kampala’s slums. In other words, Kampala’s forcibly displaced youth face high risk of HIV exposure not only due to the high prevalence of HIV in the community, but due to many factors that compound this risk.

By the same token, condom use (a demonstrably effective prevention strategy against STIs and HIV) is affected by an array of factors beyond the availability of condoms and individuals’ decisions to use them. For instance, studies have highlighted how intrapersonal (e.g., condom self-efficacy) and socio-cultural (e.g., stigma and gender norms) factors influence consistent condom use among displaced persons [37, 38]. In particular, condom self-efficacy—a key component of consistent condom use that include an individual’s knowledge, intentions, and relationship dynamics that facilitate condom negotiation [27, 39–41]—is associated with intended and actual condom use [37] and reduced sexual risk practices [42]. Unfortunately, condom self-efficacy among displaced youth remains markedly understudied, leaving interventionists and policymakers without key information for effectively promoting a useful SRH care resource among this high-risk population.

Socio-cultural factors such as stigma also constitute a significant barrier to SRH care for youth in humanitarian contexts that impedes their access to SRH services [12, 43, 44]. Adolescent SRH-related stigma (i.e., social, cultural, and religious norms associated with adolescent’s sexual practices and utilization of SRH services) is an emerging area of concern [12, 45]. For instance, a 2017 study exploring SRH-related stigma experienced by Tanzanian youth found that anticipated and perceived SRH-related stigma significantly decreased these youth’s likelihood of attempting to access SRH services [46]. Similarly, a cross-sectional study among displaced youth living in urban slums in Kampala, Uganda found that adolescent SRH stigma was associated with reduced HIV testing among girls [12]. Further, stigma is shown to act as a major barrier to STI service uptake. For instance, a 2016 systematic review [10] and a 2020 cross-sectional study [47] both found that stigma was a frequently reported barrier to young people in low-income countries accessing STI services. Moreover, a growing body of evidence has particularly emphasized the role of HIV-related stigma as a barrier to accessing HIV services. A systematic review of 10 studies with a total of 3,788 participants found that in low- and middle-income countries, high perceived levels of HIV-related stigma were twice as likely to be associated with late presentation of HIV care as were low perceived levels [48]. A more recent review highlighted how HIV-related stigma was associated with lower rates of HIV testing, reduced utilization of HIV care, and reduced adherence to HIV antiretroviral therapy [49]. In and beyond Uganda’s slums, the intersection of SRH- and HIV-related stigma may operate as powerful socio-cultural barriers to prevention, treatment uptake, and improved health outcomes. Intersecting stigma, the interaction between systems of power and oppression—such as those based on race, gender, and HIV serostatus—produce and reproduce inequity (e.g., racism, sexism, and HIV-related stigma) [50]. Qualitative research with urban refugee youth in Uganda reveal intersecting stigmas—including inequitable gender norms, HIV-related stigma, sex work stigma, and refugee stigma—shape engagement with HIV testing [43]. Less studies have specifically explored HIV-related and adolescent SRH stigma and condom self-efficacy outcomes, yet the ways in which these stigmas operate to produce shame and blame toward sexually active persons can reduce comfort and confidence discussing, purchasing, and using condoms. For instance, research with non-refugee populations in South Africa found that condom-related shame was associated with condom refusal [51], and sexual stigma was linked with lower condom self-efficacy in Jamaica [52]. The potential linkages between stigma and condom self-efficacy warrants further attention with displaced youth living in urban slums.

Studies suggest that gender norms (i.e., social and cultural expectations about women’s and men’s roles and responsibilities) are another socio-cultural factor that, like stigma, plays an important role in shaping the SRH resources and sex-related practices of a community. Substantial evidence indicates that gender norms constitute a community-level factor critical to the development and implementation of targeted HIV prevention strategies [53–57]. For instance, inequitable gender norms are shown to be associated with sexual practices that elevate HIV exposure (e.g., condomless sex) and intimate partner violence [55–57]. Such inequitable gender norms may also hinder women’s ability to refuse sexual advances or negotiate for safer sexual practices (e.g., condom use), and prompt men to not use condoms to avoid being considered unmasculine [58, 59]. By contrast, a study of adolescents in South Africa found that belief in equitable gender norms and gender roles was significantly associated with support for condom use, condom self-efficacy, and intent to use [60, 61]. The same study found that adolescent girls who supported equitable gender norms reported significantly higher rates of condom use during their last sexual encounter. Yet despite the fact that inequitable gender norms, attitudes, and practices remain entrenched in many societies [53–56], including Uganda [28], to date, only a limited number of studies have investigated the association between gender norms and condom self-efficacy and use.

### The present study

The present study seeks to address these several interrelated knowledge gaps regarding the co-occurrence of socio-cultural factors associated with condom self-efficacy and consistent condom use among displaced youth living in urban slum settings in Uganda. Guided by Tsai’s conceptualization of the synergistically interacting socio-cultural factors [31], our study seeks to understand how synergistic effects of adverse socio-cultural factors (i.e., beliefs in harmful inequitable gender norms and high adolescent SRH stigma and perceived HIV stigma) influence condom self-efficacy and practices. Our study hypotheses that: (1) we will observe more frequent co-occurrence of beliefs in harmful inequitable gender norms, high adolescent SRH, and perceived HIV stigma; (2) we will observe unique profiles of adverse socio-cultural exposures and reduced condom practices (i.e., low condom self-efficacy; condomless sex); (3) the joint effect of beliefs in harmful inequitable gender norms, high adolescent SRH, and perceived HIV stigma will decrease condom self-efficacy and practices; and (4) safer sexual communication (i.e., discussion of safer sexual practices with partner) will be a protective factor associated with increased condom self-efficacy, while condom self-efficacy and safer sexual communication will be protective factors associated with increased odds of reporting consistent condom use. Evidence shows that safer sex practices are critical to promoting condom self-efficacy and use among populations generally, and among displaced youth specifically [40, 62].

## Methods

### Sampling and data collection

This community-based cross‐sectional study was implemented in collaboration with refugee-serving non-profit agencies (i.e., Interaid Uganda, Young Africans for Integral Development, Tomorrow Vijana) and government agencies (i.e., Office of the Prime Minister, Uganda AIDS Control Program, Ministry of Health) in Uganda from January 2018 to March 2018 in five informal urban settlements (i.e., Kabalagala, Rubaga, Kansanga, Katwe, and Nsambya) in Kampala, Uganda.

To recruit participants and administer the tablet-based survey developed by our research team, we hired and trained 12 peer research assistants (PRAs) (i.e., four young men [two men from the Democratic Republic of the Congo, one from Burundi, and one from South Sudan] and eight young women [two women from Burundi, four from the Democratic Republic of the Congo, one from South Sudan, and one from Rwanda]) who self-identified as refugees or displaced persons aged 18–24 years. PRAs also received training in research ethics and methods, participant recruitment, and survey implementation, and assisted with the refinement of survey measures to enhance their appropriateness for and clarity within the local context and forcibly displaced population. The research coordinator—this paper’s lead-author—provided daily supervision to ensure data quality and adherence to data collection procedures and to address any distress from participants.

We used purposive, non‐random sampling methods to access this marginalized population [63]. PRAs used snowball sampling of their social networks to recruit forcibly displaced young women and men aged 16–24 years who (a) self-identified as a refugee or displaced person or as having refugee/displaced parents; (b) lived in one of five informal settlements (i.e., Kabalagala, Rubaga, Kansanga, Katwe, and Nsambya); (c) spoke English or Swahili and (d) were able to provide informed consent. To supplement PRA recruitment of forcibly displaced youth, we included modified peer-driven recruitment sampling methods. Namely, PRAs invited participants to recruit from 2 to 5 participants from their own social networks.

PRAs administered a structured 35–45-min tablet-based survey in English or Swahili in a location chosen by the participants (e.g., community center, football field, collaborating community agency). Participants received 12,500 Ugandan shillings (approximately $3.75) to complete the survey. All sensitive questionsincluding questions about sexual practices—were completed by participants in private. After completing the survey, all participants received information on sexual education, HIV, and psychosocial resources. Research ethics approval was obtained from the University of Toronto (#35,405) and Uganda’s Ministry of Health Ethics boards (ADM: 105/261/01).

### Measures

#### Outcome measures

*Recent consistent condom use* was only completed by participants who indicated to be sexually active, and measured with a single question: “In the last 3 months, how frequently did you or your sexual partner(s) use a condom during sexual intercourse?” Responses included: never, infrequently, sometimes, fairly often, and every time. This variable was recoded to a dichotomous outcome with participants indicating using condoms fairly often and every time coded as consistent condom users, and those who reported to never, infrequently, and sometimes using condoms coded as infrequent condom users.

*Condom self-efficacy* was assessed with the Condom Efficacy scale (Cronbach’s α = 0.95, range 7–35) [64, 65], completed by all participants regardless of their sexual activity to gain insight into their potential future behaviors and identify areas where education or intervention may be necessary. All seven items were measured on a 5-point response scale by which respondents rated their level of confidence in their ability to correctly use condoms and perceived self-efficacy in ability to ensure condoms are used in sexual encounters. The items were summed to create a cumulative condom self-efficacy score, with a lower score indicating lower levels of condom self-efficacy and higher scores indicating higher levels of condom self-efficacy. The 7-item scale has been used in studies among refugee youth [28, 47, 66], and among other vulnerable populations. [40, 52, 67]

#### Syndemic socio-cultural factors

We assessed participants' level of exposure to three socio-cultural factors: adolescent SRH stigma, perceived HIV-related stigma, and gender norms.

*Adolescent SRH (A-SRH) stigma* was assessed using the 14-item Ugandan A-SRH Stigma scale (validated and shown to be reliable in Ugandan contexts) [12], which focuses on two subscales of (a) adolescents’ sexual activity and pregnancy and (b) adolescent modern family planning and abortion stigma (Cronbach’s α = 0.74). Response options were provided on a 3-point Likert scale (i.e., disagree, neutral, agree) that coded all “agree” responses as “1,” with higher summed response scores reflecting higher levels of stigma. We used a median split to dichotomize A-SRH with participants whose summed responses totaled greater than 8 were coded as “1” and classified as having high A-SRH stigma.

*Perceived HIV-related stigma* was assessed using a 10-item stigma subscale (Cronbach α = 0.87) [68]. Higher scores indicated the presence of higher perceived HIV-related stigma. Following the distribution, sums of scores were dichotomized using a median cut-off of > 32, indicating high perceived HIV-related stigma, which was coded as “1.” This scale has previously been used with displaced youth living in urban slums in Uganda [69].

*Belief in harmful inequitable gender norms* were assessed using the gender belief scale (Cronbach α = 0.76). Responses include “strongly agree,” “agree,” “disagree,” and “strongly disagree” [69]. We reverse coded the items, and summed them up so that high scores represented more traditional beliefs about gender roles and low scores indicated low progressive beliefs. We then used a median split to create a dichotomous variable with values > 26 indicating that a participant personal beliefs in harmful inequitable gender norms. This scale has previously been used with displaced youth living in urban slums in Uganda [28].

#### Protective factors

S*afer sex communication* was assessed using a single question: “How often do you suggest practicing safer sex with your sexual partner(s)?” and sexual history communication was measured based on participants’ response to the question, “How often do you ask your sexual partner(s) about their sexual history before having sex?” Response sets each protective factor were measured as: never (1); sometimes (2); usually (3); or always (4).

#### Covariates

Sociodemographic variables included age, education level (i.e., no education/less than secondary school, and post-secondary education), employment status (i.e., employed, unemployed, and student), and gender (i.e., young women/young men).

### Data analysis

We first conducted descriptive analyses for all variables for the entire sample. Bivariate analyses were performed to identify differences of socio-demographic characteristics by sexual intercourse history. Second, we calculated co-occurrence of adverse socio-cultural factors by summing the variables and calculating the percentage of occurrences for each participant. Third, adjusting for sociodemographic factors (i.e., age, gender, and employment), we tested sets of multivariable logistic (binary outcomes) models and multivariate regression (continuous outcomes) models to create unique profiles of forcibly displaced youth based on beliefs in harmful inequitable gender norms, high adolescent SRH, perceived HIV-related stigma, and condom condom self-efficacy and consistent condom use. Several regression analyses were conducted, and when a variable was the outcome, it was excluded as an independent measure. Finally, we tested for synergistic interactions among socio-cultural factors using both multivariable logistic (binary outcome: recent consistent condom use) and multivariate (continuous outcome: condom self-efficacy) regression models. Two-way interactions between beliefs in harmful inequitable gender norms, high adolescent SRH, and perceived HIV-related stigma were included in the model. First, we adjusted for sociodemographic variables; in the final model of condom self-efficacy, we added protective factors of safer sexual communication; and in the models of recent consistent condom use, we added condom self-efficacy and safer sex communication. In this way, we tested the role of protective factors in the associations between syndemics adverse socio-cultural factors and condom self-efficacy and practices. All regression models were adjusted for age (in years), gender, education, and employment.

## Results

### Sample characteristics

Table 1 shows the characteristics of the sample and how participants differed by sexual history. Most of the sample consisted of young women (*n* = 333; 74.8%), with a mean age of 19.59 years. Over two thirds (*n* = 278; 62.5%) of participants reported having had sex in their lifetime, with 53.3% (*n* = 133) reporting having recently consistently used condoms during sex. Differences by ever having had sex were found for age, education level, employment status, condom self-efficacy. Participants who had received more than a secondary school education were more likely to report ever having had sex. Participants’ condom self-efficacy scores ranged from 7 to 35, with a mean of 19.33 (SD = 7.83). Differences for condom self-efficacy were significant (*p* = 0.006), suggesting participants’ perceptions of their ability to use condoms varied by sexual history. Participants who had ever had sex reported a higher condom self-efficacy score (*M* = 20.12; SD = 8.05) than those who had never had sex (*M* = 18.02; SD = 7.29).

**Table 1:** Characteristics of forcibly displaced youth living in the slums of Kampala, Uganda by sexual history (*N* = 445)

Variables	Full sample (*N* = 445)	Ever had sex (*n* = 278; 62.5%)	Never had sex (*n* = 167; 37.5%)	*p*-value
	*N* (%)/Mean (SD, range)	*n* (%)/Mean (SD, range)	*n* (%)/Mean (SD, range)	
Age	19.59 (2.59, 16–24)	20.54 (2.48, 16–24)	18.02 (1.94, 16–24)	0.001
Gender				
Young women	333 (74.8)	201 (60.4)	132 (39.6)	0.113
Young men	112 (25.2)	77 (68.8)	35 (31.3)	
Education				0.001
Less than secondary school	234 (52.6)	120 (43.2)	114 (68.3)	
Secondary school education	211 (47.4)	158 (56.8)	53 (31.7)	
Place of birth				0.051
South Sudan	35 (7.9)	16 (5.8)	19 (11.4)	
Burundi	115 (25.8)	67 (24.1)	48 (28.7)	
Democratic Republic of the Congo	249 (56.0)	169 (60.8)	80 (47.9)	
Rwanda	19 (4.3)	12 (4.3)	7 (4.2)	
Others	27 (6.1)	14 (5.0)	13 (7.8)	
Immigration status (*n* = 442)				0.723
Refugees	391 (88.5)	243 (88.0)	148 (89.2)	
Seeking asylum/undocumented	51 (11.5)	33 (12.0)	18 (10.8)	
Employment status (*n* = 428)				0.001
Student	190 (44.39)	76 (28.4)	114 (71.3)	
Employed	62 (14.49)	59 (22.0)	3 (1.9)	
Unemployed	176 (41.12)	133 (49.6)	43 (26.9)	
Condom self-efficacy	19.33 (7.83, 7–35)	20.12 (8.05, 7–35)	18.02 (7.29, 7–35)	0.006

### Co-occurrence of adverse socio-cultural factors

As illustrated in Table 2, among the 445 forcibly displaced youth, irrespective of other adverse socio-cultural factors, about 81.6% reported experiencing and holding high A-SRH stigma. Close to half of the participants reported perceiving high levels of HIV-related stigma (41.6%) and personal beliefs in harmful inequitable gender norms (46.3%). We also observed a significantly higher proportion of girls compared to boys reporting high perceived HIV-related stigma (45.9% vs. 28.6%; *p* = 0.001) and personal beliefs in harmful inequitable gender norms (55.9% vs. 17.9%; *p* = 0.001). Table 2 reports the co-occurrence of personal beliefs in harmful inequitable gender norms, high A-SRH stigma, and perceived HIV-related stigma. Overall, 42.73% (*n* = 190) reported two co-occurring socio-cultural factors, ranging from to 1.35% (*n* = 6; co-occurrence of beliefs in harmful inequitable gender norms and perceived HIV-related stigma) to 20.90% (*n* = 93; co-occurrence of A-SRH stigma and perceived HIV-related stigma). About 16.63% (*n* = 74) of participants reported co-occurrence of the three factors, while only 6.52% (*n* = 29) reported no adverse socio-cultural factors.

**Table 2:** Adverse socio-cultural factors among forcibly displaced youth (*N* = 445): Inequitable gender norms, adolescent SRH stigma, perceived HIV stigma, and their co-occurrence

Adverse socio-cultural factors and their combinations	Full sample (*N* = 445), *N* (%)	Adolescent boys and young men (*n* = 112), *N* (%)	Adolescent girls and young women (*n* = 333), *N* (%)	X^2^
A-SRH stigma				0.01
Low	82 (18.4)	21 (18.8)	61 (18.3)	
High	363 (81.6)	91 (81.3)	272 (81.7)	
Perceived HIV Stigma				10.42***
Low	260 (54.1)	80 (71.4)	180 (54.1)	
High	185 (41.6)	32 (28.6)	153 (45.9)	
Beliefs in harmful inequitable gender norms				48.67***
Low	239 (53.7)	92 (82.1)	147 (44.1)	
High	206 (46.3)	20 (17.9)	186 (55.9)	
Each exposure only				
Beliefs in harmful inequitable gender norms only	35 (7.87)			
A-SRH stigma only	105 (23.60)			
HIV stigma only	12 (2.70)			
Co-occurrence of adverse socio-cultural exposures				
Beliefs in harmful inequitable gender norms + A-SRH stigma	91 (20.45)			
Beliefs in harmful inequitable gender norms + HIV stigma	6 (1.35)			
A-SRH stigma + HIV stigma	93 (20.90)			
Beliefs in harmful inequitable gender norms + A-SRH stigma + HIV stigma	74 (16.63)			
No adverse socio-cultural exposures	29 (6.52)			

*p < 0.05; **p < 0.01; ***p < 0.001

### Associations between adverse socio-cultural factors and condom practice variables

We created separate profiles of displaced youth who reported adverse socio-cultural factors (i.e., Beliefs in harmful inequitable gender norms, A-SRH stigma, and perceived HIV-related stigma) and condom self-efficacy and practices (Table 3). Beliefs in harmful inequitable gender norms (β = -0.16; *p* < *0.0*10) and A-SRH stigma (β = − 0.11; *p* < *0.0*10) were significantly associated with decreased condom self-efficacy, while recent consistent condom use (β = 0.28; *p* < *0.0*01) was associated with increased condom self-efficacy. There was a bi-directional relationship between A-SRH stigma (adjusted odds ratio [aOR] = 2.75; 95% confidence interval [CI] [1.35, 5.63]) and perceived HIV-related stigma (aOR = 2.85; 95% CI [1.39, 5.85]). High condom self-efficacy reduced personal beliefs in harmful inequitable gender norms (aOR = 0.94; 95% CI [0.90, 0.98]) and A-SRH stigma (aOR = 0.95; 95% CI [0.90, 0.99]), and increased recent consistent condom use (aOR = 1.12; 95% CI [1.07, 1.17]).

**Table 3:** Examining intersections between beliefs in inequitable gender norms, adolescent SRH stigma, perceived HIV-related stigma, and condom self-efficacy and practice variable

Independent variables	BIGN^δ^ (aOR, 95% CI)	A-SRH stigma (aOR, 95% CI)	Perceived HIV stigma (aOR, 95% CI)	Condom self-efficacy (β, 95% CI)	Consistent condom use (AOR, 95% CI)
BIGN^δ^		0.87 (0.44, 1.73)	0.80 (0.44, 1.47)	− 0.16 (− 4.42, − 0.91)**	0.69 (0.36, 1.31)
A-SRH stigma	0.91 (0.45, 1.83)		2.85 (1.39, 5.85)**	− 0.11 (− 3.96, − 0.17)**	0.84 (0.41, 1.73)
Perceived HIV stigma	0.78 (0.42, 1.44)	2.75 (1.35, 5.63)**		0.03 (− 1.14, 2.24)	0.74 (0.40, 1.38)
Condom efficacy	0.94 (0.90, 0.98)**	0.95 (0.90, 0.99)*	1.01 (0.97, 1.06)		1.12 (1.07, 1.17)***
Consistent condom use	0.75 (0.39, 1.44)	0.86 (0.42, 1.74)	0.73 (0.39, 1.35)	0.28 2.84, 6.31)***	

Covariates include age, gender, and education level

*p < 0.05; **p < 0.01; ***p < 0.001

^δ^Beliefs in harmful in inequitable Gender Norms

### Model of synergistic interactions between adverse socio-cultural factors and condom self-efficacy and practice variables

#### Condom self-efficacy

We used multivariate linear regression models with product terms to estimate the multiplicative interaction for adverse socio-cultural factors on condom self-efficacy (Table 4). The joint effect of beliefs in harmful inequitable gender norms with high A-SRH stigma (Model 1: β = − 0.20; *p* < 0.05) and high A-SRH stigma with high perceived HIV-related stigma (Model 3: β = − 0.31; *p* < 0.001) were associated with reduced condom self-efficacy. When all two-way product terms (adjusting for age, gender, education) were entered into the regression model (i.e., Model 4 in Table 4), we found a multiplicative interaction for joint effects of high A-SRH stigma and high perceived HIV-related stigma (β = − 0.29; *p* < 0.05) on condom self-efficacy. When protective factors of safer sexual communication and sexual history discussions were introduced in the model, we found that safer sexual communication (Model 5: β = 0.26; *p* < 0.001) acted as a protective factor on condom self-efficacy. The joint effect of high A-SRH stigma and high perceived HIV-related stigma was not significant when the protective factors were introduced in the model.

**Table 4:** Multiplicative two-way interactions of adverse socio-cultural factors and protective factors on condom self-efficacy (*N* = 445)

Variables and product term	Model 1: BIGN^δ^ X A-SRH stigma (β)	Model 2: BIGN X HIV stigma (β)	Model 3: A-SRH stigma X HIV stigma (β)	Model 4: All two-way product terms (β)	Model 5: All two-way product terms, main effect and protective factors (β)
*Product terms*					
BIGN^δ^ X A-SRH stigma	− 0.20*			.17	0.14
BIGN X HIV stigma		− 0.01		.01	− 0.15
A-SRH stigma X HIV stigma			− 0.31***	− 0.29*	− 0.26
*Protective factors*					
Sexual history discussions					0.05
Safer sexual communication					0.26***

Covariates include sociodemographic factors: age, gender, and education

*p < 0.05; ** *p* < 0.01; *** *p* < 0.001

^δ^Beliefs in harmful inequitable gender norms

#### Recent consistent condom use

Table 5 shows logistic regression models with product terms to assess the multiplicative effects of adverse socio-cultural factors on recent consistent condom use. We found a multiplicative interaction for a joint effect of high A-SRH stigma with high perceived HIV-related stigma (Model 3: aOR = 0.52; 95% CI [0.28, 0.96]) on recent consistent condom use. In a logistic regression model including all two-way product terms and controlling for age, gender, and employment (i.e., Model 4 in Table 5), we found that a multiplicative interaction for joint effects of high A-SRH stigma and high perceived HIV-related stigma on recent consistent condom use was still significant. When we introduced protective factors in Model 5, we found that none of the joint effects were significant and that condom self-efficacy (aOR = 1.01; 95% CI [1.05, 1.16]) and safer sexual communication (aOR = 2.12; 95% CI [1.54, 2.91]) acted as protective factors on inconsistent condom use.

**Table 5:** Multiplicative two-way interactions of adverse socio-cultural factors and protective factors on recent consistent condom use among a sample of sexually active forcibly displaced youth living in the slums of Kampala, Uganda (*N* = 243)

Variables and product term	Model 1: BIGN^δ^ X A-SRH stigma (aOR, 95% CI)	Model 2: IGN X HIV stigma (aOR, 95% CI)	Model 3: A-SRH stigma X HIV stigma (aOR, 95% CI)	Model 4: All two-way product terms (aOR, 95% CI)	Model 5: All two-way product terms with protective factors (aOR, 95% CI)
BIGN X A-SRH stigma	0.64 (.35, 1.78)			0.58 (0.28, 1.23)	0.78 (0.26, 6.25)
BIGN X HIV stigma		0.66 (0.31, 1.41)		1.68 (0.54, 5.23)	1.75 (0.49, 6.21)
A-SRH stigma X HIV stigma			0.52 (0.28, .96)*	0.45 (0.03, .64)*	0.57 (0.24, 1.35)
*Protective factors*					
Condom self-efficacy					1.01 (1.05, 1.16)***
Sexual history discussion					0.85 (0.59, 1.24)
Safer sexual communication					2.12 (1.54, 2.91)***

Covariates include sociodemographic factors: age, gender, and education

*p < 0.05; **p < 0.01; ***p < 0.001

^δ^ Beliefs in inequitable Gender Norms

## Discussion

In this community-based cross-sectional study among displaced youth recruited from five urban informal settlements in Kampala, Uganda, we observed (1) that adverse sociocultural factors (i.e., Beliefs in harmful inequitable gender norms, high A-SRH stigma, and perceived HIV-related stigma) co-occurred, and (2) that these adverse socio-cultural factors interacted synergistically to decrease condom self-efficacy and recent consistent condom use). For example, we found a joint effect of personal beliefs in inequitable gender norms with high A-SRH stigma, and of high A-SRH stigma with high perceived HIV-related stigma, on reduced condom use self-efficacy. Further, we found that the multiplicative interaction of high A-SRH stigma and high perceived HIV stigma was associated with reduced recent consistent condom use. However, it is worth noting that this association disappeared when protective factors of condom self-efficacy and safer sexual communication are introduced in the model. Taken together, these findings suggest that interventions tailored to displaced youth that seek to reduce intersecting stigmas and promote gender equity have the potential to *also* improve condom use self-efficacy—an exceptionally impactful additional outcome. Thus, our findings provide a critical empirical foundation to inform future multilevel HIV prevention interventions for the growing number of displaced populations living in urban slums globally.

Prior studies have shown that experiences of adverse socio-cultural factors by youth in humanitarian contexts can affect their access to and utilization of HIV prevention services [12, 43, 44]. In our sample of displaced youth living in urban slums, about half (42.73%; *n* = 190) reported experiencing two or more co-occurring adverse socio-cultural factors, which is notably higher than rates identified in Singer and colleagues’ [70] scoping review (12%; *n* = 22) and in Mendenhall and Singer’s [36] review (stigma: *n* = 19; 32%) of the literature with non-forcibly displaced samples. It is plausible that youth’s adverse socio-cultural experiences prior to displacement may continue and be amplified by their belief of harmful norms in their new settlement post-displacement. To better tailor and target SRH interventions for displaced youth living in urban slums, future studies should assess the extent to which the effects of adverse socio-cultural norms on youth SRH practices are amplified by the shocks associated with forcible displacement and the norms endemic to refugee communities.

Although very few studies have examined the association between stigma and sexual practices that elevate HIV exposure among refugees, our findings indicate that HIV-related stigma and A-SRH stigma jointly were associated with reduced condom self-efficacy and reduced recent consistent condom use. Among sexually active displaced youth, those who reported higher levels of A-SRH and perceived HIV stigma were less likely to report recent consistent condom use. It is plausible that participants who experience intersectional stigma maybe ashamed and blamed for being sexually active, which in turn reduces their comfort and confidence discussing, purchasing, and using condoms. Experiences of condom-related shame could negatively influence individuals’ decision when and why to use condoms [51], and lower their condom self-efficacy [52]. Indeed, youth’s belief in harmful norms regarding their sexual activity may co-occur [49] with HIV-related stigma experiences, thereby compromising a youth’s confidence in their ability to influence their behaviors, environment, and future [49]. This finding underscores the need for incorporating adolescent-responsive stigma-reduction programs into sexual health interventions for displaced youth, and the utility of applying a syndemics framework to assess the interactions between social and health disparities to optimally tailor such interventions [36, 71]. Specifically, there is a need to work to reduce youth’s stigmas towards SRH and HIV, and reduce their belief in harmful inequitable gender norms.

We also found that condom self-efficacy was a protective factor that weakened the detrimental joint effect that HIV-related stigma and A-SRH stigma had on recent consistent condom use. These findings align with prior findings that condom self-efficacy served as a protective factor against the negative effect of sexual stigma on condom use among men who have sex with men in Jamaica [52]; that adolescent girls and women in Ghana who reported higher levels of SRH stigma were less likely to have used modern contraception [45]; and that displaced youth living in urban slums in Uganda who reported high levels of SRH stigma tended to exhibit lower HIV testing awareness and uptake [12]. Yet the present study goes beyond these analyses by highlighting the protective role of condom self-efficacy on the (potentially compounding) impact of *intersecting* stigma on consistent condom use. By revealing the potential for condom self-efficacy in improving sexual health outcomes, the present study provides valuable insights for SRH interventions, particularly those serving populations exposed to socio-cultural factors shown to inhibit condom use and engagement with other SRH services.

Our findings that (a) belief in inequitable gender norms were associated with reduced condom self-efficacy among the entire sample and that (b) condom self-efficacy was a protective factor for consistent condom use among sexually active youth extends prior findings that belief in inequitable gender norms reduced youth’s likelihood of supporting condom use, having condom use self-efficacy, and intending to use condoms [60, 61]. Namely, our study identified a joint effect of belief in inequitable gender norms and A-SRH on condom self-efficacy, but no relation on recent consistent condom use. The joint effect of these two adverse socio-cultural factors may hinder the development of condom self-efficacy across intrapersonal (knowledge about using condoms), interpersonal (condom negotiation skills), and structural (cost, access to condoms) dimensions [58, 59]. Our syndemic analyses of these two socio-cultural factors underlines the need for HIV prevention interventions to address both personal belief in inequitable gender norms and stigma targeting adolescents in order to promote their uptake of needed SRH services effectively.

Our promising findings have multiple implications for future HIV prevention research and programming. Gender transformative interventions (i.e., interventions that actively engage men, challenge gender norms, and address power inequities between genders) [72] must not only center gender equality in the development and implementation of these interventions, but also target HIV vulnerability at individual, interpersonal, and community levels, with careful attention to socio-cultural factors that may affect program engagement. Among displaced youth living in urban slums in Uganda specifically, these interventions should provide context-responsive strategies to challenge the intersectional stigma they face, given our findings that these intersecting stigma can powerfully inhibit these youth’s engagement with SRH services and health-promoting behaviors [13, 43]. With safer sex communication and condom self-efficacy emerging as protective factors against syndemic adverse socio-cultural factors, there is a need for condom negotiation interventions [73]. These interventions should concurrently address technical communication (i.e., HIV information; where to get condoms) and transformative communication (i.e., how to negotiate condom use with partners; sex-positive approaches to condom use) because they are critical to increasing consistent condom use [74]. Such interventions could apply multi-media approaches and offer opportunities to practice condom negotiation skills.

At the community level, few interventions designed to reduce intersectional stigma exist beyond the pilot stage in low- and middle-income countries [75, 76]. Therefore, HIV prevention researchers and practitioners should take steps to adapt and implement multi-component stigma-reduction interventions that address intersecting stigmas related to gender, age, and refugee status as well as HIV-related stigma and SRH stigma. Displaced populations commonly experience other stigmas (e.g., related to ethnicity, displaced status), and other vulnerabilities (e.g., poverty) that negatively impact their SRH. Therefore, stigma-reduction interventions could simultaneously address multiple intersecting stigmas (e.g., HIV-related and A-SRH stigma) and multiple targets of stigma (i.e., individuals, health care facilities, communities, and policies). Such efforts will require the development and coordination of multiple and multi-level strategies such as increasing individuals’ peer social support networks and knowledge regarding SRH practices and resources, training staff and instituting protocols at health care facilities, and empowering community leaders to address stigma [76].

Our study’s promising findings should be considered in light of several limitations. First, our results cannot be generalized to all forcibly displaced youth, given that this study used a non-randomized cross-sectional sample of displaced youth living in urban five slums in Kampala. Therefore, similar longitudinal studies should be conducted using randomized samples to test the generalizability of our findings. Second, although this study, like most sexual health studies with adolescents, used self-reported data, these data may be influenced by recall bias or social desirability bias. For instance, we use a single consistent condom use measure, and other measures that have not been validated among forcibly displaced youth in sub-Saharan Africa. Future studies should culturally validate measures used in this study, test the moderating role of partner sexual communication characteristics on consistent condom use among displaced youth living in urban slums and examine other HIV prevention strategies such as pre-exposure prophylaxis and the dapivirine vaginal ring. Also, further research is needed to understand a broader range of factors impacting safe-sex practices, including intersections with other vulnerabilities. Third, although our study explored socio-cultural factors associated with condom use, future studies need to explore other barriers to condom use such as cost, negative attitudes, reduced sexual pleasure, fit-and-feel problems and erection difficulties. Fourth, our study did not explore age and gender differences due to being under powered. Therefore, future studies should consider conducting age and gender disaggregated analyses to understand the unique factors that influence condom self-efficacy and practices so as to inform age-appropriate and gender-specific interventions.

## Conclusions

Despite these limitations, our study has several strengths. First, we build on the scant literature examining how socio-cultural factors are associated with condom self-efficacy and practices among displaced youth living in urban slums. To our knowledge, our study is the first to identify how syndemic interactions of adverse socio-cultural exposures influence condom self-efficacy and practices among displaced youth living in urban slums. Second, we found a joint effect between inequitable gender norms and A-SRH that was negatively associated with condom self-efficacy, highlighting the importance of applying a gender transformative lens [72] in sexual health interventions for displaced youth living in urban slums. Third, we found that safer sexual communication (i.e., partner discussing safer sexual practices) was a protective factor in the relationship between synergistically interacting stigmas (i.e., HIV-related and A-SRH-related stigma) and condom self-efficacy. This finding builds on the existing literature by elucidating the compounding effect of syndemic adverse socio-cultural factors on condom self-efficacy and practices among forcibly displaced youth. We also found that condom self-efficacy and safer sexual communication acted as protective factors in the relationship between intersectional stigma and recent consistent condom use, which underscores the utility of multi-faceted HIV prevention approaches that address both intersectional stigma and relationship dynamics among displaced youth living in urban slums. These findings also signal the need for HIV prevention interventions tailored to displaced youth living in urban slums to target both community-level (intersecting stigma, equitable gender norms) and interpersonal-level (safe sexual communication) and intrapersonal-level (condom self-efficacy) factors to improve condom use among individuals. In particular, our study emphasizes that gender transformative approaches and intersectional stigma interventions are urgently needed to improve the sexual health of the ever increasing forcibly displaced populations [2].

## Data Availability

The data that support the findings of this study are available upon reasonable request from the corresponding author. The data are not publicly available due to research ethics board restrictions.

## Abbreviations

aOR: Adjusted odds ratio
A-SRH: Adolescent sexual and reproductive health
CI: Confidence interval
HIV: Human immunodeficiency virus
IGN: Inequitable gender norms
PRA: Peer research assistant
SRH: Sexual and reproductive health
STI: Sexually transmitted infections
